# Theorizing that Psychedelic Assisted Therapy May Play a Role in the Treatment of Trauma-Induced Personality Disorders

**Published:** 2024-11-15

**Authors:** Gianni Martire, Daniel Sipple, David Baron, Mark S. Gold, Kai-Uwe Lewandowski, Catherine A. Dennen, Alireza Sharafshah, Igor Elman, Panayotis K. Thanos, Edward J. Modestino, Rajendra D. Badgaiyan, Albert Pinhasov, Abdalla Bowirrat, Milan Makale, A. Kenison Roy, Keerthy Sunder, Kevin T. Murphy, Shaurya Mahajan, Yatharth Mahajan, Chynna Levin, Kenenth Blum

**Affiliations:** 1 Biophysics Research in Advanced Interdisciplinary Neuroscience at Applied Physics, New York, USA; 2 Midwest Brain and Spine Institute, Roseville, USA; 3 Division of Addiction Research, Therapy and Education, Center for Sports, Exercise and Mental Health, Western Health Sciences, Pomona, USA; 4 Department of Psychiatry, Stanford University School of Medicine, Palo Alto, USA; 5 Department of Psychiatry, Washington University School of Medicine, St. Louis, USA; 6 Division of Personalized Pain Therapy Research & Education, Center for Advanced Spine Care of Southern Arizona, Tucson, USA; 7 Department of Orthopaedics, Fundación Universitaria Sanitas Bogotá D.C. Colombia; 8 Department of Orthopaedics, Universidade Federal do Estado do Rio de Janeiro, Brazil; 9 Department of Family Medicine, Jefferson Health Northeast, Philadelphia, USA; 10 Cellular and Molecular Research Center, School of Medicine, Guilan University of Medical Sciences, Rasht, Iran; 11 Cambridge Health Alliance, Harvard Medical School, Cambridge, MA, USA; 12 Department of Molecular Biology, Adelson School of Medicine, Ariel University, Ariel, Israel; 13 Department of Pharmacology and Toxicology, Jacobs School of Medicine and Biosciences, State University of New York at Buffalo, Buffalo, USA; 14 Brain & Behavior Laboratory, Department of Psychology, Curry College , Milton, MA., USA.; 15 Department of Psychiatry, Texas Tech University Health Sciences, School of Medicine, Lubbock, USA; 16 Department of Radiation Oncology, University of California San Diego, La Jolla, CA, USA; 17 Department of Psychiatry, Tulane University School of Medicine, New Orleans, LA., USA; 18 Karma Doctors & Karma TMS, and Suder Foundation, Palm Springs, CA, USA; 19 Department of Medicine, University of California, Riverside School of Medicine, Riverside, CA, USA; 20 Division of personalized neuromodulation, Peak Logic, LLC., Del Mar, CA., USA; 21 The Blum Neurogenetic & Behavioral Institute, LLC., Austin, USA

**Keywords:** Borderline personality disorder, Post-traumatic stress disorder, Psychedelic assisted therapy, Trauma, Psilocybin

## Abstract

Borderline personality disorder (BPD) and post-traumatic stress disorder (PTSD) share overlapping neurobiological mechanisms particularly reward deficiency and stress-like anti-reward processes. And so, BPD may be reclassified as a “traumatic personality stress disorder” (TPSD) with ensuing common therapeutic strategies that may stabilize dopaminergic reward function such as psychedelic-assisted therapy. Integrated therapeutic strategies may be further supported by genetic studies aimed at assessing similarities between the two therapeutic entities. In this perspective we theorize that psychedelic assisted therapy (PAT) may play a role in the treatment of trauma induced personality disorders. This study identifies PAT as a pathway for treating both BPD and PTSD, proposing that reframing BPD as TPSD could lead to more effective, personalized interventions, ultimately improving the quality of life for those affected by trauma. Such a reclassification might also mitigate stigma, enhance our understanding of the underlying mechanisms, and optimize therapeutic interventions for a broader range of diagnostic categories characterized by anhedonia, negative affective states, hypervigilance, and dissociation.

## Introduction

BPD is a mental illness that severely impacts a person’s ability to manage their emotions. This loss of emotional control can increase impulsivity, affect how a person feels about themselves, and negatively impact their relationships with others. Effective treatments are available that can help people manage the symptoms of BPD. In clinical settings, the prevalence is around 10% to 12% in outpatient psychiatric clinics and 20% to 22% among inpatient clinics [1]. BPD often occur with other mental illnesses, such as PTSD. These co-occurring disorders can make it harder to correctly diagnose and treat BPD especially when the disorders have overlapping symptoms. For example, a person with BPD also may be more likely to experience symptoms of major depression, PTSD, bipolar disorder, anxiety disorders, substance use disorder (SUD) or even eating disorders like binge eating.

PTSD is a mental health condition that’s caused by an extremely stressful or terrifying event-either being part of it or witnessing it. Symptoms may include flashbacks, nightmares, severe anxiety and uncontrollable thoughts about the event. Most people who go through traumatic events may have a hard time adjusting and coping for a short time. But with time and by taking good care of themselves, they usually get better. If the symptoms get worse, last for months or years, and affect their ability to function daily, they may have PTSD. It has been estimated that 3.9% of the world population has had PTSD at some stage in their lives. Moreover, most people exposed to potentially traumatic events do not develop PTSD, and more women are affected by PTSD than men [2].

The growing body of evidence is increasingly highlighting the potential of psychedelic-assisted therapy in mitigating symptoms across a broad spectrum of psychiatric disorders [3]. Classic hallucinogens, also known as psychedelics, primarily function as 5-HT2A receptor agonists. They alter brain activity and connectivity, induce shifts in cognitive and conscious states, and are especially notable for enhancing cognitive flexibility, fostering neuroplasticity, and modifying cognitive processes [4].

Conversely, compounds like 3,4-Methylenedioxymethamphetamine (MDMA) have pronounced psychotropic effects. MDMA inhibits the reuptake of serotonin, dopamine, and norepinephrine, increasing their availability and eliciting feelings of openness, trust, and social connectivity [5].

Extensive clinical trials have investigated the safety and efficacy of MDMA-assisted therapy for PTSD treatment. Phase 3 trial results have consistently shown substantial reductions in PTSD symptoms compared to placebo controls [6–8]. While long-term follow-up data remain limited, existing studies indicate that the positive effects on PTSD symptoms persist, with observations continuing at 1 and 3.5 year intervals [9–11]. However, it is noteworthy that the FDA recently rejected the use of MDMA to treat PTSD. The FDA pointed out that the available evidence failed to show that the drug is effective or that its benefits outweigh its risks [12].

MDMA assisted therapy typically is generally considered safe. Documented side effects are usually mild to moderate, encompassing symptoms like teeth grinding, headache, and restlessness. Most of these adverse events subside soon after the treatment ends. Crucially, MDMA microdosing has not been linked to abuse potential, suicidality, or QT prolongation. In fact, while these doses of MDMA are well tolerated [9–11], its use outside of a tightly controlled therapeutic setting can pose significant risks. In one study involving doses ranging from 25 mg, plus 12.5 mg supplemental dose or full-dose MDMA at 125 mg, plus 62.5 mg supplementary dosing did not see statistically significant reductions in clinician-administered PTSD scale (CAPS) scores (p = 0.066), although there was clinically and statistically significant self-reported (PDS) improvement (p = 0.014). CAPS scores improved further in the 1-year follow-up. In addition, three MDMA sessions were more effective than two (p = 0.016) [9]. In fact, microdosing of psychedelics is a growing phenomenon that has shown benefits in some preclinical data; however, a recent self-directed controlled trial reported no evidence of improved mood [13]. The accumulating evidence underscores the safety and efficacy of psychedelic compounds as novel therapeutic agents in psychiatry [14, 15].

### Psilocybin-assisted therapy in MDD and TRD

Extensive research has consistently demonstrated the efficacy of psilocybin-assisted therapy in alleviating symptoms in patients with major depressive disorder (MDD) and treatment-resistant depression (TRD) [16–21]. Psilocybin, classified as a classic psychedelic, acts as a 5HT2A receptor agonist [4]. When it binds to this receptor, psilocybin triggers a cascade of psychotropic effects, including perceptual alterations, enhanced introspection, and increased emotional openness [3, 4]. Moreover, it stimulates the release of BDNF, fosters neurogenesis, and enhances neuroplasticity, all of which significantly contribute to the psychotherapeutic process [22, 23].

The emerging body of evidence substantiates the claim that psilocybin notably reduces depressive symptoms in individuals with MDD and TRD [16, 19]. Additionally, research indicates that psilocybin can improve mental health outcomes for those experiencing anxiety [24]. While many studies assessing the effectiveness of psilocybin for psychiatric disorders lack a placebo group, alternative control conditions such as escitalopram, low-dose psilocybin, and waitlists have been utilized [16, 17]. The current scarcity of phase 3 clinical trials is mirrored by a limited number of reported adverse events related to psilocybin use. Nonetheless, the preliminary data available suggest that psilocybin-assisted therapy is generally safe [25, 26].

### Potential role of psilocybin in treating bpd symptoms

The potential role of psilocybin in treating PTSD is drawing increasing attention, with several leading institutions like Halucenex Life Sciences Inc., COMPASS Pathways, Combat Stress, Johns Hopkins University, and Ohio State University at the forefront of research. These organizations are conducting phase 3 clinical trials, primarily focusing on recruitment stages, with outcomes yet to be published. Given the positive therapeutic responses observed with both psilocybin and MDMA-despite their different mechanisms of action-and the established effectiveness of MDMA-assisted therapy in mitigating PTSD symptoms [4, 10], there is a reasoned hypothesis that psilocybin-assisted therapy could be similarly beneficial for PTSD.

Psilocin, the active metabolite of psilocybin and a hallucinogenic component of *Psilocybe mexicana,* is structurally analogous to serotonin (5-HT). Sakashita et al. [27] studied the effects of psilocin on dopamine and 5-HT concentrations in specific brain regions of awake rats. Systemic administration of psilocin significantly increased extracellular dopamine levels in the nucleus accumbens, while 5-HT levels remained unchanged in this area. Conversely, in the medial prefrontal cortex, psilocin markedly elevated extracellular 5-HT levels but reduced dopamine levels. Behaviorally, psilocin notably increased head twitches in rats. These findings suggest that psilocin can elevate both dopamine and 5-HT concentrations in the mesoaccumbens and/or mesocortical pathways [28–32].

Specifically, psilocin (3-[2-(dimethylamino)ethyl]-1*H*-indol-4-ol) is a hallucinogenic component of the Mexican mushroom *Psilocybe mexicana* and a skeletal serotonin (5-HT) analogue. treating BPD is critical. Sakashita et al. [27], examined the effects of systemically administered psilocin on extracellular dopamine and 5-HT concentrations in the ventral tegmental area (VTA), nucleus accumbens, and medial prefrontal cortex of the dopaminergic pathway in awake rats using *in vivo* micro dialysis. Intraperitoneal administration of psilocin (5, 10 mg/kg) significantly increased extracellular dopamine levels in the nucleus accumbens. Psilocin did not affect the extracellular 5-HT level in the nucleus accumbens. Conversely, systemic administration of psilocin (10 mg/kg) significantly increased extracellular 5-HT levels in the medial prefrontal cortex of rats, but dopamine was decreased in this region. Behaviourally, psilocin significantly increased the number of head twitches. Thus, data suggest that psilocin increased both the extracellular dopamine and 5-HT concentrations in the mesoaccumbens and/or mesocortical pathway [27]. In terms of the importance of stabilizing dopamine there are 394 articles listed in PUBMED retrieved on [10–23, 24, 33–37].

Considering the overlapping mechanism and symptomatology of PTSD and BPD, psilocybin presents as a potential treatment option for BPD. Its ability to enhance emotional regulation, encourage introspection, and modify neural circuits essential to emotional processing positions it as a promising therapeutic candidate for BPD. When integrated with specific psychotherapeutic approaches, psilocybin-assisted therapy may offer significant benefits in managing emotional dysregulation and related symptoms in BPD patients. Due to its apparent potential comprehensive evaluation of psilocybin’s effectiveness in treating BPD is critical. It is also possible that other forms of PAT such as Ketamine etc. for example could potentially be utilizable in terms of potential treatment benefits [38].

### Integration of PTSD/BPD trauma, reward pathway, and psychedelic assisted therapy

#### PTSD/BPD trauma

There is some evidence to support the concept that both PTSD and BPD share similar unwanted events especially related trauma. Specifically, De Jongh et al. [28] revealed that an intensive trauma-focused treatment is a feasible and safe treatment for PTSD patients with clinically elevated symptoms of BPD, and that BPD symptoms decrease along with the PTSD symptoms. In fact, Powers et al. [29] used exploratory structural equation modeling (ESEM) to replicate and extend prior work by including two models to examine how PTSD (ICD-11, DSM-5), and BPD symptoms relate. They reported a three-factor ESEM model of complex PTSD (ICD-11 PTSD symptoms) and BPD symptoms that best fit the data and observed support for discriminant validity between factors across trauma-related avoidance, aggressive behavior, and anxious attachment. For DSM-5 PTSD, a two-factor ESEM model was best-fitting (PTSD and BPD). Moreover, Vera Békés et al. [39] attempted to establish the relationship between childhood trauma, moral injury (MI), and BPD, PTSD, and disturbances in self-organization symptoms, a core diagnostic criterion of complex PTSD besides PTSD symptoms, and shame as a moral emotion in an inpatient psychiatric sample. They found that their results pinpoint the importance of considering MI as a critical factor of patient experiences in relation to childhood trauma that potentially contributes to the development of psychiatric symptoms [30]. In addition, Sachsse et al. [31] evaluated the results of a specific psychodynamically oriented trauma-focused inpatient treatment for women with complex PTSD and concomitant BPD, self-mutilating behavior, and depression. Following one-year, the frequency of inpatient treatments (hospitalizations) decreased dramatically.

We encourage the interested reader to scrutinize the recent review by Leichsenring et al. [32]. Accordingly, BPD is characterized by instability of self-image, interpersonal relationships and poor effect. Moreover, symptoms include impulsivity, intense anger, feelings of emptiness, strong abandonment fears, suicidal or self-mutilation behavior, and transient stress-related paranoid ideation or severe dissociative symptoms. The disorder is associated with considerable functional impairment, intensive treatment utilization, and high societal costs. The risk of self-mutilation and suicide is high. In the general adult population, the lifetime prevalence of BPD has been reported to be from 0.7 to 2.7%, while its prevalence is about 12% in outpatient and 22% in inpatient psychiatric services. BPD is significantly associated with other mental disorders, including depressive disorders, SUD, PTSD, attention-deficit/hyperactivity disorder, bipolar disorder, bulimia nervosa, and other personality disorders. Compared to treatment as usual, psychotherapy has proved to be more efficacious, with effect sizes between 0.50 and 0.65 with regard to core BPD symptom severity. As pointed out by Leichsenring et al. [32], there is no evidence available consistently showing that any psychoactive medication is efficacious for the core features of BPD. This is why we are proposing the potential use of psychedelic-assisted therapy.

Moreover, based on a review of the literature from 2000 to 2023 genetic factors account for 40–60% of BPD variation, with significant roles played by epigenetic alterations like DNA methylation and microRNAs, particularly in the context of childhood trauma. Gene-environmental interactions are also vital for BPD’s development. Treatments such as dialectical behavior therapy, mentalization-based therapy, and schema therapy have shown efficacy, with success variability possibly linked to genetic factors. The take home message is that the promise of personalized medicine in BPD treatment, driven by genetic insights is indeed the future of appropriate novel treatment for not only bpd but PTSD [40].

## Conclusion

We are proposing that both BPD and PTSD share overlapping neurobiological mechanisms particularly reward deficiency and stress-like anti-reward processes. We believe based on current evidence that BPD potentially could be reclassified as a “TPSD” with ensuing common therapeutic strategies that may stabilize dopaminergic reward function such as psychedelic-assisted therapy. We theorize that PAT following intensive research might play a role in the treatment of trauma-induced personality disorders. This study suggests PAT as a potential pathway for treating both BPD and PTSD, proposing that reframing BPD as TPSD could lead to more effective, personalized interventions, ultimately improving the quality of life for those affected by trauma. It is indeed plausible that reclassification could also mitigate stigma, augment our comprehension of the underlying mechanisms, and optimize therapeutic interventions for a broader range of diagnostic categories characterized by negative affective states, hypervigilance, anhedonia, and dissociation.
